# Design and preliminary evaluation of the FINGER rehabilitation robot: controlling challenge and quantifying finger individuation during musical computer game play

**DOI:** 10.1186/1743-0003-11-10

**Published:** 2014-02-04

**Authors:** Hossein Taheri, Justin B Rowe, David Gardner, Vicki Chan, Kyle Gray, Curtis Bower, David J Reinkensmeyer, Eric T Wolbrecht

**Affiliations:** 1 Mechanical Engineering Department, University of Idaho, Moscow, ID, USA; 2 Department of Biomedical Engineering, University of California, Irvine, CA, USA; 3 Department of Rehabilitation, University of California, Irvine, CA, USA; 4 Department of Mechanical and Aerospace Engineering, University of California, Irvine, CA, USA; 5 Department of Anatomy and Neurobiology, University of California, Irvine, CA, USA

**Keywords:** Robotic rehabilitation, Stroke, Motor control, Mechanism synthesis, Finger individuation, Color-based motion capture

## Abstract

**Background:**

This paper describes the design and preliminary testing of FINGER (Finger Individuating Grasp Exercise Robot), a device for assisting in finger rehabilitation after neurologic injury. We developed FINGER to assist stroke patients in moving their fingers individually in a naturalistic curling motion while playing a game similar to Guitar Hero^®^^a^. The goal was to make FINGER capable of assisting with motions where precise timing is important.

**Methods:**

FINGER consists of a pair of stacked single degree-of-freedom 8-bar mechanisms, one for the index and one for the middle finger. Each 8-bar mechanism was designed to control the angle and position of the proximal phalanx and the position of the middle phalanx. Target positions for the mechanism optimization were determined from trajectory data collected from 7 healthy subjects using color-based motion capture. The resulting robotic device was built to accommodate multiple finger sizes and finger-to-finger widths. For initial evaluation, we asked individuals with a stroke (n = 16) and without impairment (n = 4) to play a game similar to Guitar Hero^®^ while connected to FINGER.

**Results:**

Precision design, low friction bearings, and separate high speed linear actuators allowed FINGER to individually actuate the fingers with a high bandwidth of control (−3 dB at approximately 8 Hz). During the tests, we were able to modulate the subject’s success rate at the game by automatically adjusting the controller gains of FINGER. We also used FINGER to measure subjects’ effort and finger individuation while playing the game.

**Conclusions:**

Test results demonstrate the ability of FINGER to motivate subjects with an engaging game environment that challenges individuated control of the fingers, automatically control assistance levels, and quantify finger individuation after stroke.

## Background

Over the past several decades, researchers have developed robotic devices for rehabilitation therapy after stroke. This is in response to a sizable need, with nearly 800,000 people per year suffering a stroke in the United States alone [1]. Of the survivors, approximately two-thirds experience long-term impairment of their affected upper-extremity [2]. Robotic therapy devices can automate the repetitive and strenuous aspects of conventional physical therapy. Furthermore, robotic therapy devices can serve as scientific instruments for quantifying the recovery process, and thus may provide insight that is not normally available with conventional therapy alone.

Robot assisted therapy of the upper extremity following stroke has been shown to be as effective as, and in some cases modestly more effective than, conventional therapy (for reviews see [3-7]). Research with robotic therapy devices supports the contention that motor recovery increases with therapy intensity [6], i.e. more practice is better. What remains unclear, however, is how a rehabilitation robot should interact with the patient in order to optimize recovery during practice. One approach is to help patients practice movements that they cannot complete without assistance, which may foster somatosensory stimulation that induces brain plasticity [8]. Indeed, most rehabilitation robots are strong enough to complete movements even when patients are completely impaired and/or when tone and spasticity act in opposition. However, care must be taken so that the robot does not “take over” the movement practice from the patient, which may cause the patient to “slack” and reduce their effort at the task being practiced [9,10]. Patient effort is considered crucial to increasing motor-plasticity during rehabilitation therapy [11,12]. Thus, it seems important for robotic rehabilitation devices to simultaneously enable movement practice and encourage patient effort during therapy.

Numerous control strategies for robotic therapy have been successfully implemented and tested, as summarized in [13]. Of specific interest are “assist-as-needed” control strategies, which change assistance in response to perceived effort, typically correlated in some way to performance error (tracking error or similar). These controllers alter the assistance level by modifying controller parameters (e.g. feedback gains, desired trajectory shape and/or timing, model based terms, etc.) [9,14-18]. Tests with these controllers suggest that increased error encourages patient effort, and vice-versa, although the relationship remains unclear. Additional experiments may clarify this and other relationships affecting motor recovery during rehabilitation therapy, although the ultimate validation clearly depends on therapeutic efficacy.

Effectively exploring the factors that promote functional recovery during movement therapy and evaluating “assist-as-needed” and other control strategies depends on the control fidelity of the robotic platform. To quantify baseline motor ability, ideally, the robot should be able to appear both massless and frictionless to the patient, and should be highly compliant and backdriveable. However, it is also important to have a high bandwidth of force control, as to not limit the response of robot during interaction with the patient. Improving the control and impedance characteristics of a rehabilitation robot has the potential to make such devices better scientific instruments as well as allowing more precise investigation of motor learning and the mechanisms of neuroplasticity, as suggested by [13].

Another critical consideration for understanding the mechanisms by which rehabilitation robots promote recovery is the limb of application of the robot. As an integral part of activities of daily living (ADLs), rehabilitation of the hand is particularly important, and a significant need exists for improved hand rehabilitation, as most of those who have suffered a stroke experience some impairment in hand function [19]. Furthermore, the hand and fingers have a highly developed neuro-muscular system to which the brain has dedicated a large portion of resources.

Designing a robot to actuate the hand or finger is a significant challenge, as evidenced by the large variety of robotic devices that have been developed for hand and finger therapy. Previous work has focused often on re-creating the complexity of hand and finger movements, often at the expensive of actuation and control. These hand robots typically fall into the category of end-effector or exoskeleton (for review see [20]). End-effector devices attach distally and do not attempt to align with the joint axis of the patient, as exoskeleton devices typically do.

In the work presented here, we sought to maximize controller fidelity and minimize the mechanical impedance of the device, at the expense of the robot’s degrees-of-freedom. Although each finger in the human hand has multiple degrees-of-freedom, most ADLs incorporate a simple finger curling motion, similar to a power grasp [21]. Thus, an opportunity existed to create a finger-curling robot with one degree-of-freedom, high control fidelity, and low friction.

FINGER, the finger curling robot presented here, is capable of individually assisting both the index and middle fingers through a natural grasping motion (Additional file 1). Each finger is individually guided by an 8-bar mechanism that controls the orientation and position of the proximal phalanx and the position of the middle phalanx. Each 8-bar mechanism has a single degree-of-freedom and is actuated by a high bandwidth and low-friction linear electric actuator. Further friction reduction is achieved through feed-forward control compensation.

In the sections that follow, we present the design, controller development, and preliminary testing of FINGER. We present the mechanism synthesis, which is based on motion capture of finger grasping motion, first. We then describe the mechanical design, including sizing adjustments and patient-robot interface. In the third section, we describe the actuation including controller development and friction compensation. Finally, we present some results from pilot testing with several subjects who have suffered a stroke. Portions of this work have appeared previously in conference paper format [22,23].

## Methods

### Mechanism design

#### ***Finger curling data acquisition and analysis***

This section describes motion capture and data analysis used to characterize a basic finger curling motion, similar to a power grasp [21]. Although the human hand can perform many differing grasps and grips in order to manipulate objects, the basic curling motion is the most common and therefore we focused on it for administering and studying finger movement therapy.

**Figure 1:**
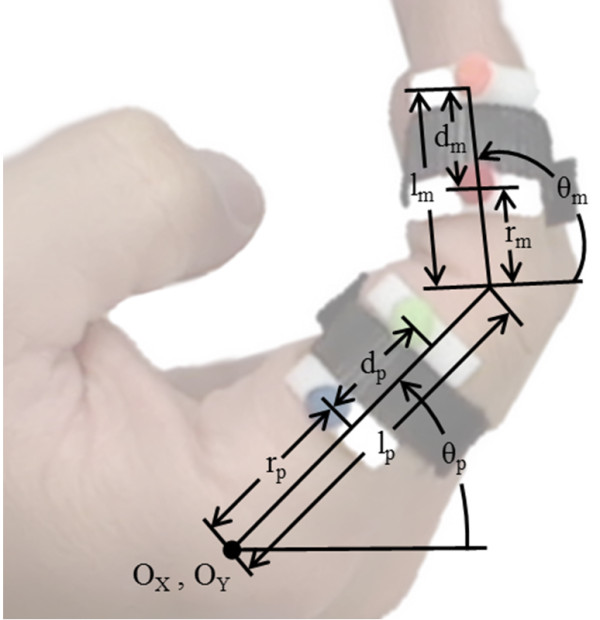
The setup and dimensions collected from motion capture and regression.

Color-based motion tracking [24] was used to record the path of the index finger as the hand performed a curling motion. Subjects’ hands were filmed with a single camera from above, where the index and middle fingers curled in a plane perpendicular to the camera. Any out-of-plane motion during curling was small and treated as noise. The back of the hand was placed against a rest aligning it with the x-axis. Four brightly colored felt dots of differing colors were attached to the index finger, two each on the center-lines of the proximal and middle phalanges, which are the defined attachment points for the 8-bar mechanism. Likewise, the colored dots were attached using hook-and-loop straps that were the same thickness as the planned mechanism straps. The placement of the felt dots allowed the centerlines of the proximal and middle phalanges to be recorded throughout the curling motion. See Figure 1.

Seven healthy adult subjects were asked to curl their hand, meeting the thumb in a circle, for a minimum of 10 times. They were not given any further instructions regarding how to curl their hand, in order to produce the most natural motion. Dimensions of the index and middle fingers and hand were recorded for each subject using calipers. The lengths of the proximal and middle phalanges for the index and middle fingers were recorded in a similar fashion as [25]. The distances between creases for both the index and middle fingers were also recorded in the same manner as [26].

The path of the four felt dots was regressed against a planar, two revolute joint model, shown in Figure 1. The regression model determines 5 dimensions using the system of equations given below in (1). These equations are defined for each frame of the motion capture.

(1) $$\left[\begin{array}{ccccc} 1 & 0 & c_{p} & 0 & 0\\ 0 & 1 & s_{p} & 0 & 0\\ 1 & 0 & c_{p} & 0 & 0\\ 0 & 1 & s_{p} & 0 & 0\\ 1 & 0 & 0 & c_{m} & c_{p}\\ 0 & 1 & 0 & s_{m} & s_{p}\\ 1 & 0 & 0 & c_{m} & c_{p}\\ 0 & 1 & 0 & s_{m} & s_{p} \end{array}\right]\left[\begin{array}{c} O_{x}\\ O_{y}\\ r_{p}\\ r_{m}\\ l_{p} \end{array}\right]=\left[\begin{array}{c} {\left(p_{1}\right)}_{x}\\ {\left(p_{1}\right)}_{y}\\ {\left(p_{2}\right)}_{x}-d_{p}c_{p}\\ {\left(p_{2}\right)}_{y}-d_{p}s_{p}\\ {\left(m_{1}\right)}_{x}\\ {\left(m_{1}\right)}_{y}\\ {\left(m_{2}\right)}_{x}-d_{m}c_{m}\\ {\left(m_{2}\right)}_{y}-d_{m}s_{m} \end{array}\right],$$

where

$$\begin{array}{ll} c_{p}=\left({\left(p_{2}\right)}_{x}-{\left(p_{1}\right)}_{x}\right)/d_{p} & s_{p}=\left({\left(p_{2}\right)}_{y}-{\left(p_{1}\right)}_{y}\right)/d_{p}\\ c_{m}=\left({\left(m_{2}\right)}_{x}-{\left(m_{1}\right)}_{x}\right)/d_{m} & s_{m}=\left({\left(m_{2}\right)}_{y}-{\left(m_{1}\right)}_{y}\right)/d_{m}\\ d_{p}=||p_{2}-p_{1}|| & d_{m}=||m_{2}-m_{1}||. \end{array}$$

In (1) above, **m**_1_, **m**_2_, **p**_1_ and **p**_2_ are the positions of the four markers, and *O*_*x*_ and *O*_*y*_ define the location of the metacarpophalangeal (MCP) joint of the index finger. During mechanism design, this point is assumed to be the origin. The length of the proximal phalanx is denoted *l*_*p*_ and the final two parameters are the distances to the proximal and middle strap attachment from the previous joint, referred to as the proximal, *r*_*p*_, and middle, *r*_*m*_, radii. It may seem that these radii should center the straps along the proximal and middle phalanges, but in practice the position along the proximal phalanges that is most comfortable for a strap is significantly forward of the center of the phalanx. For example, in Figure 1 it can be seen that the hook and loop strap holding the felt dots to the proximal phalanx sits comfortably at more than half the distance along the proximal phalanx from the MCP joint. The same relationship is true for the middle phalanx.

The mean length of the index finger proximal phalanx determined by the motion capture and regression analysis was 42 mm, with a standard deviation of 3 mm. This mean value was compared to [25] which contains a statistical analysis of 4000 hand samples. The ratio of this mean proximal length to the mean proximal length reported in [26] was multiplied by the standard deviation also reported in [26], producing a scaled standard deviation 3 mm, which is close to the standard deviation of the small data sample used.

Following the approach in [27], the length change of the proximal phalanx between successive mechanism sizes was chosen to be two standard deviations. By scaling the other variables accordingly, the complete dimensions for the other finger sizes may be found, as given in Table 1. This range of finger sizes provides an acceptable coverage of the population of hand sizes.

**Table 1:** Dimensions determined for different hand sizes

	***l***_***p ***_**(mm)**	***r***_***p ***_**(mm)**	***r***_***m ***_**(mm)**
Extra-small	28.68	16.28	10.78
Small	35.13	20.32	13.21
Medium	41.58	24.57	15.63
Large	48.03	28.40	18.06

The dimension *l*_*p *_is the proximal length, *r*_*p *_is the proximal radius, and *r*_*m *_is the middle radius.

This approach to regression has the advantage of determining the dimensions of the finger independent of the motion type. Thus, the angular relationship between the phalanges can be used independently to define the finger motion. Specifically, regression is used to determine the middle phalanx angle, *θ*_*m*_, as a function of the proximal phalanx angle, *θ*_*p*_, using a second-order polynomial equation.

**Figure 2:**
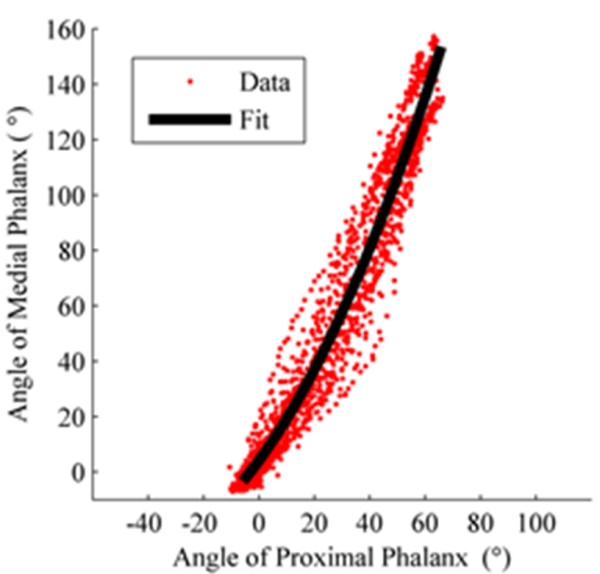
**The angular relationship between the middle and proximal phalanges during finger curling.** The red points were collected from motion capture, and the black line is the regressed quadratic curve-fit.

Figure 2 shows the relationship between the middle phalanx angle and the proximal phalanx angle for the curling motion collected from all 7 motion capture subjects. The black line is a quadratic curve-fit. With significant variance in hand sizes, the relationship between the two angles appears uncorrelated to hand size.

The 2nd order equation used to fit the data as shown in Figure 2 is:

(2) $$\theta_{m}=0.77660232\theta_{p}{}^{2}+1.37397306\theta_{p}+0.07324267.$$

This equation and finger dimensions given in Table 1 were used to generate the 15 target points for each mechanism size, consisting of 15 desired points and angles of the proximal phalanx and 15 desired points of the middle phalanx, repeated for each of three sizes: extra-small, small, medium, and large. This number of points was chosen in order to maintain a good spatial resolution while keeping computational complexity of the design reasonable. The target points were created using the 2 revolute joint planar model with the angle of proximal phalanx, *θ*_*p*_, varied from 0° to 60° discretized into 15 evenly spaced target angles. The angle of the middle phalanx, *θ*_*m*_, was determined from (2) and the target points for both the proximal and middle phalanges were defined at 19 mm behind the center-line of the finger to allow for a means of connecting the robot to the hand.

#### ***Linkage selection***

Designing a mechanism to reach multiple end-effector configurations, known as mechanism synthesis, is a well-studied research area [28]. This particular application, however, has a unique twist. In this case, there is not a single desired configuration but rather two that are correlated; one for the proximal phalanx (position and angle), and one for the middle phalanx (position only). Furthermore, the design specifies a planar grasping motion with a single DOF for each finger. Planar mechanisms, with their multiple varieties of single DOF configurations, provide an adequate solution base for this design problem.

**Figure 3:**
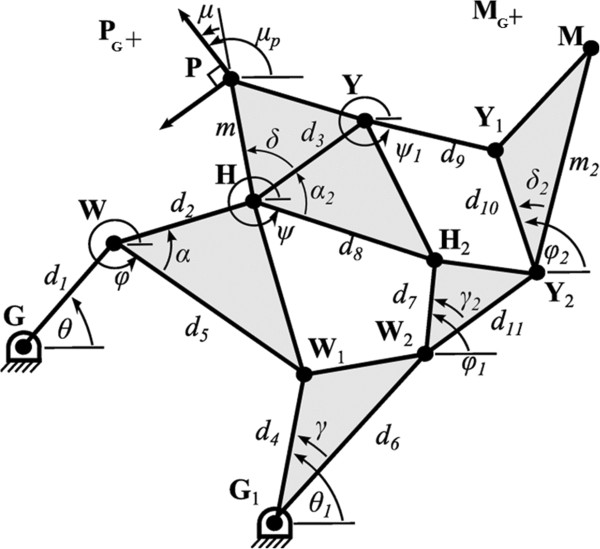
**Structural dimensions and configuration angles of the 8-bar mechanism.** Reproduced from [23] with permission from IEEE. Goal positions for the proximal and middle phalanges are shown as P_*G *_and M_*G*_, respectively. The goal angle of the proximal phalanx is *μ*_*p*_.

Initial mechanism synthesis attempts explored multiple configurations of Watt type six-bar chains [29], but were ultimately unsuccessful in reproducing the desired output configuration. The final design uses an eight-bar mechanism (Chain 1 from [29]) with revolute joints (see Figure 3). The goal configurations consist of the position (**P**_*G*_) and angle (*μ*_*P*_) of the proximal phalanx and the position of the middle phalanx (**M**_*G*_). The mechanism is made up of 10 revolute joints (**G**, **G**_1_, **W**, **W**_1_, **W**_2_, **H**, **H**_2_, **Y**, **Y**_1_, and **Y**_2_) and 7 links defined by the kinematic chains **GW**, **WHW**_1_, **G**_1_**W**_1_**W**_2_, **HPYH**_2_, **W**_2_**H**_2_**Y**_2_, **Y**_1_**Y**_2_, and **YMY**_1_. These links are defined by seven structural angles (*α*, *α*_2_, *δ*, *δ*_2_, *γ*, *γ*_2_, and *μ*) and 13 structural lengths (*d*_1-11_, *m*, and *m*_2_). Figure 3 also shows the seven configuration angles (*θ*, *θ*_1_, *ϕ*, *ϕ*_1_, *ϕ*_2_, *ψ*, and *ψ*_1_) that changes as the mechanism moves. The mechanism has 1 DOF so that specifying one of these configuration angles specifies the complete configuration of the mechanism.

The preliminary optimization of this mechanism was presented in [23]. The approach here is similar, but here the mechanism configuration has changed so that the middle phalanx end-effector is connected to link **Y**_1_**Y**_2_ rather than **YY**_1_. This change improved the ability of the optimization process to reach desired middle phalanx target points, and also made the resulting mechanism easier to manufacture.

#### ***Mechanism design equations***

The design equations that define the 8-bar mechanism consist of both path and loop equations. The path equations consist of three separate kinematic paths through the mechanism from one of the fixed pivots to the each of the end effectors (**P** and **M**). The path equations are similar to those presented in [23] but have changed to improve optimization and manufacturability, based on a trial and error design process. For the proximal phalanx, the shortest path equation is:

(3) $$G+d_{1}\left\{\begin{array}{c} c\theta \\ s\theta \end{array}\right\}+d_{2}\left\{\begin{array}{c} c\left(\alpha +\phi \right)\\ s\left(\alpha +\phi \right) \end{array}\right\}+m\left\{\begin{array}{c} c\left(\psi +\alpha_{2}+\delta \right)\\ s\left(\psi +\alpha_{2}+\delta \right) \end{array}\right\}=P,$$

where *c* and *s* stand for cosine and sine, respectively, and the other parameters are previously defined and shown in Figure 3. The other two path equations for the proximal phalanx are defined by the kinematic chains **G**_1_**W**_2_**H**_2_**HP** and **G**_1_**W**_2_**Y**_2_**Y**_1_**YHP**. As previously mentioned, the middle phalanx path equations have changed more significantly. Using the same notation, the shortest path to the middle phalanx is

(4) G1+d6cθ1sθ1+d11cφ1−γ2sφ1−γ2+m2cφ2−δ2sφ2−δ2=M,

The other two kinematic paths to the middle phalanx are defined by the kinematic chains **G**_1_**W**_2_**Y**_2_**M** and **GWHYY**_1_**Y**_2_**M**. The design equations also include 3 internal loop constraint equations which must be satisfied to keep the design viable. The inner loop equation is

(5) $$G+d_{1}\left\{\begin{array}{c} c\theta \\ s\theta \end{array}\right\}+d_{5}\left\{\begin{array}{c} c\varphi \\ s\varphi \end{array}\right\}-d_{4}\left\{\begin{array}{c} c\theta_{1}\\ s\theta_{1} \end{array}\right\}=G_{1}.$$

The other two equations, for the middle and outer loops are defined by the kinematic chains **GWHH**_2_**W**_2_**G**_1_ and **GWHYY**_1_**Y**_2_**W**_2_**G**_**1**_.

As mentioned before, the design specifies a goal angle of the proximal phalanx, *μ*_*P*_ , in addition for each goal position of the proximal phalanx, **P**_*g*_. Using the relationship

(6) $$\psi =\mu_{P}-\alpha_{2}-\delta -\mu ,$$

the goal angle for the proximal phalanx is substituted into the previously presented path and loop equations to constrain the configuration angle *ψ* to the goal angle of the proximal phalanx, *μ*_*P*_, and the structural angles *α*_2_, *δ*, and *μ*.

With the path and loop equations defined, the design problem becomes a function minimization problem. The objective is to find the structural variables and the set of *n* = 15 configuration angles that best reach the 15 desired configurations. To achieve this, a cost function is created consisting of the sum of the squares of the distance between the desired end-effector points **P** and **M** and the goal positions **P**_*G*_ and **M**_*G*_ for each of the 15 desired configurations. The cost function is defined

(7) $$J=\overset{15}{\underset{n=1}{\sum }}\left({\left(P_{n}-P_{G,n}\right)}^{T}\left(P_{n}-P_{G,n}\right)+{\left(M_{n}-M_{G,n}\right)}^{T}\left(M_{n}-M_{G,n}\right)\right),$$

where **P**_*n*_ is the position of point **P** at angle *θ*_*n*_ and **P**_**G**,*n*_ is the *n*^*th*^ desired configuration (with similar definitions for **M** and **M**_*G*_). Only one configuration angle is necessary as the mechanism has only 1 DOF, and *θ* was arbitrarily chosen (the other possibility was *θ*_1_), even though in the final design we selected *θ*_1_ as the input angle for connecting the actuator (based on the locations of **G** and **G**_1_).

#### ***Mechanism design equation constraints***

In addition to the cost function, a large set of constraints are required based on the overall design goals and manufacturing considerations. Our preliminary approach to consstraints was presented in [23]. Some constraints require only upper and lower bounds, such as the location of the base points, **G** and **G**_1_, and structure variables *d*_1 − 11_, *m*, and *m*_2_. Other constraints require additional calculations, such as the link dimensions not specifically specified by the structural dimensions (the distance ${\overset{¯}{\mathrm{HW}}}_{1}$, for example). These constraints are summarized, with brief explanations, in Table 2.

**Table 2:** Mechanism structural design constraints

**Dimension(s)**	**Bounds (mm)**	**Purpose**
**G**_*x*_, **G**_1,*x*_	{−76.2, 25.4}	Keep fixed pivots located behind wrist/hand.
**G**_*y*_, **G**_1,*y*_	{−10.2, 2.54}	Keep fixed pivots located behind wrist/hand.
*d*_1 − 7_	{1.91, 12.7}	Min. distance to manufacture joints, keep mechanism compact.
*d*_8 − 11_	{1.91, 7.62}	Min. distance to manufacture joints, keep mechanism compact.
*m*	{1.27, 12.7}	Min. distance to manufacture proximal phalanx end-effector, keep mechanism compact.
*m*_2_	{1.91, 12.7}	Min. distance to manufacture middle phalanx end-effector (including room for rotating joint), keep mechanism compact.
$\overset{¯}{GG_{1}}$, $\overset{¯}{\mathrm{PY}}$, ${\overset{¯}{\mathrm{HW}}}_{1}$, $\overset{¯}{W_{1}W_{2}}$, $\overset{¯}{H_{2}Y}$, $\overset{¯}{H_{2}Y_{2}}$, and $\overset{¯}{MY_{1}}$	{1.91, 7.62}	Min. distance to manufacture joints, keep mechanism compact.

These constraints govern the structural dimensions of the 8-bar mechanism, but do not limit the location of the free joints (**W**, **W**_1_, **W**_2_, **H**, **H**_2_, **Y**, **Y**_1_, and **Y**_2_) as the mechanism moves through the desired configurations. For example, any two joints should not overlap during the motion of the mechanism. This requires that the location of each joint be calculated at each of the 15 goal configurations. In total there are 29 joint pairs with the potential to overlap which are constrained to keep the distance between joints from going below a manufacturable distance. The complete list of joint-to-joint distances to calculate at each of the 15 goal configurations is given in Table 3.

**Table 3:** 8-bar mechanism joint distance constraints

**Joint-to-joint distance(s), calculated at each of the 15 goal configurations**	**Bounds (mm)**	**Purpose**
$\overset{¯}{WG_{1}}$, $\overset{¯}{W_{1}G}$, $\overset{¯}{W_{2}G}$, $\overset{¯}{W_{2}W}$, $\overset{¯}{W_{2}H}$, $\overset{¯}{\mathrm{HG}}$, $\overset{¯}{HG_{1}}$, $\overset{¯}{H_{2}G}$, $\overset{¯}{H_{2}G_{1}}$, $\overset{¯}{H_{2}W}$, $\overset{¯}{H_{2}W_{1}}$, $\overset{¯}{\mathrm{YG}}$, $\overset{¯}{YG_{1}}$, $\overset{¯}{\mathrm{YW}}$, $\overset{¯}{YW_{1}}$, $\overset{¯}{YW_{2}}$, $\overset{¯}{Y_{1}G}$, $\overset{¯}{Y_{1}G_{1}}$, $\overset{¯}{Y_{1}W}$, $\overset{¯}{Y_{1}W_{1}}$, $\overset{¯}{Y_{1}W_{2}}$, $\overset{¯}{Y_{1}H}$, $\overset{¯}{Y_{1}H_{2}}$, $\overset{¯}{Y_{2}G}$, $\overset{¯}{Y_{2}G_{1}}$, $\overset{¯}{Y_{2}W}$, $\overset{¯}{Y_{2}W_{1}}$, $\overset{¯}{Y_{2}H}$, $$\overset{¯}{Y_{2}Y}$$	{19.1, 254}	Keep joints from colliding during motion, and make joints manufacturable.

**Figure 4:**
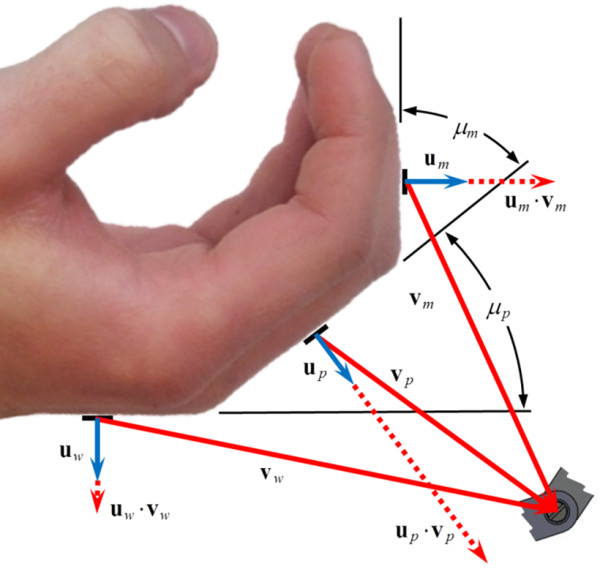
Vectors for constraining the location of the free joints to the back of the hand and finger.

One of the most important set constraints concerns the location of the joints with respect to the hand and fingers throughout the motion. One of the main goals for the mechanism was that it be entirely located behind the hand during operation. This goal was chosen to allow the hand to be easily attached to the robot, to allow stacking of mechanisms for individual fingers, and to facilitate providing sensory stimulation to the volar surface of the hand. For instance, a soft object can be mounted towards the palm of users’ hands to be touched while practicing grasp motions. In the presented experiment, however, this feature is not utilized. This requires all of the joints to be behind the hand and fingers at each of the 15 goal configurations. Thus the constraint area is constantly changing. We implemented this constraint area by creating three separate unit vectors, one at the back of the middle phalanx, **u**_*m*_, one at the back of the proximal phalanx, **u**_*p*_, and one at the back of the wrist, **u**_*w*_. These vectors are illustrated in Figure 4. These unit vectors at the phalanges point away from the hand at an angle perpendicular to the phalanx and are different for each of the 15 goal configurations. The unit vector at the back of the wrist also points away from the hand, but does not move for different goal configurations. Furthermore, three vectors are created for each of the free joints at each of the 15 goal configurations. These three vectors, **v**_*m*_, **v**_*p*_, and **v**_*w*_, all point to the aforementioned joint and originate from the base of the unit vectors **u**_*m*_, **u**_*p*_, **u**_*w*_ (see Figure 4). The maximum of the dot products between the corresponding **u** and **v** vectors gives the distance, as a positive value, that the joint is from the back of the hand/finger. This value is constrained to between 12.7 and 76.2 mm, so that the joints clear the back of the hand during motion but are also kept from begin excessively away from the hand.

#### ***Mechanism cost function minimization***

The cost function (7) was minimized within the bounds of the constraints detailed in the previous section using a constrained multivariable minimization optimizer (Matlab function “fmincon”). The minimization process was repeated numerous times with randomized initial conditions until viable solutions were found. The minimization was first performed on the goal configurations generated from the dimensions of the medium size given in Table 1. The resulting structural parameters of the medium mechanism are given in Table 4. The total error after cost function minimization was 2.14 mm for all 30 of the target points for the 15 medium finger size goal configurations. This is an average error of 0.071 mm per target point.

**Table 4:** Structural parameters for the medium finger curling mechanism

**Length (mm)**	**Angle (°)**
**G**_*x*_ = −40.93	*α* = −135.2
**G**_*y*_ = −28.68	*α*_2_ = +59.18
**G**_1*x*_ = −59.64	*δ* = +9.520
**G**_1*y*_ = −25.40	*δ*_2_ = −24.25
*d*_1_ = +36.28	*γ* = −39.29
*d*_2_ = +49.12	*γ*_2_ = 2.057
*d*_3_ = +19.05	*μ* = −19.67
*d*_4_ = +34.43	
*d*_5_ = +20.18	
*d*_6_ = +70.30	
*d*_7_ = +92.68	
*d*_8_ = +76.20	
*d*_9_ = +69.80	
*d*_10_ = +76.20	
*d*_11_ = +55.30	
*m* = +47.24	
*m*_2 _= +101.20	

**Figure 5:**
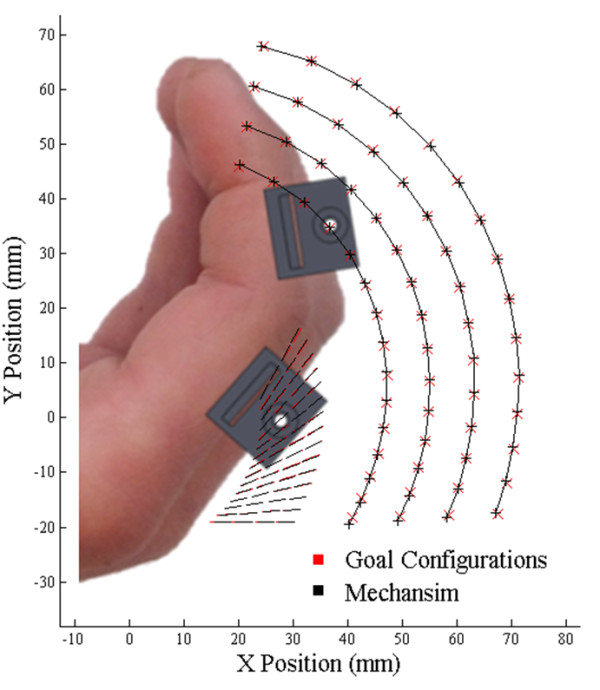
**Optimized mechanism paths four different finger sizes.** There are 15 goal configurations for each size, including target points (red exes) for the middle phalanx (for controlling position) and target lines (read lines) for the proximal phalanx (for controlling position and angle). The ability of the mechanism to reach these 15 configurations is demonstrated with black crosses and black lines.

After the structural parameters for the medium finger size were determined, the cost function was re-minimized for the large, small, and extra small finger sizes using the medium structural parameters as the initial conditions. During re-minimization, all of the structural parameters were fixed except for five (*m*, *m*_2_, *δ*, *δ*_2_, & *μ*) which allow the new mechanism it to reach the goal configurations of the other finger sizes. Although other choices of parameters also allow the minimization to reach the trajectories for the other finger sizes, changing these five parameters can be accomplished by only modifying the shape the two end-effector links. This allows the re-sizing to be accomplished without the need to disassemble the mechanism when changing between sizes. The resulting values of these structural parameters and the minimized cost function for the other mechanism sizes are given in Table 5. A visual depiction of the ability of the four different mechanism sizes to reach the four sets of 15 desired configurations is shown in Figure 5.

**Table 5:** Values of the changing structural parameters for different mechanism sizes and the resulting cost function

**Parameter**	**Extra-small**	**Small**	**Medium**	**Large**
*m* (mm)	+41.5	+44.2	+47.2	+50.1
*m*_2_ (mm)	+94.5	+67.6	+101.2	+105.7
*δ* (*°*)	+145.7	+120.6	+95.2	+74.2
*δ*_2_ (*°*)	−32.79	−28.46	−24.25	−20.45
*μ* (*°*)	−19.93	−19.99	−19.67	−19.13
Cost function_,_*J* (mm)	7.58	3.42	2.14	5.01
Per 30 points, *J*/30 (mm)	0.25	0.11	0.07	0.17

#### ***Mechanical design***

The current version of FINGER has two identical planar 8-bar mechanisms to individually curl the index and middle fingers through a naturalistic motion. The mechanism, actuators, and adjustment assemblies are located behind the hand. As mentioned above, this allows contact of the volar surface of the hand with objects during therapy, and makes it easier to attach the hand of a subject to the robot.

Each 8-bar mechanism was designed with alternating inner links and outer link pairs, overlapping at joints to balance bearing forces and keep friction low. Two ABEC 5 bearings and one precision shoulder bolt were used for each joint. The links were designed in Solidworks to the dimensions determined from the mechanism synthesis and machined from aluminum using a three-axis, computer numerical control (CNC) milling machine. The linkage design includes mechanical hard stops to limit the range of motion to the desired range.

**Figure 6:**
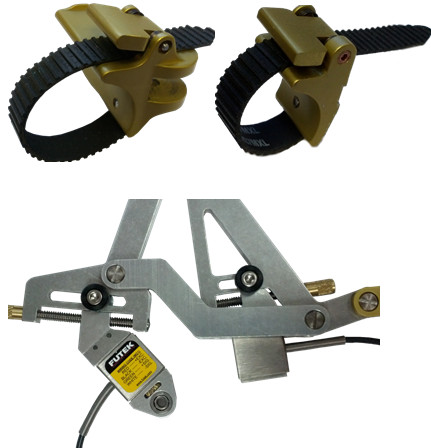
Finger cups with ratcheting straps for the middle phalanx (top left) and the proximal phalanx (top right) and finger length adjustment (bottom).

Finger cups with custom ratcheting straps (see Figure 6) are located at the two end effectors of each mechanism to attach the robot to the subjects’ proximal and middle phalanges. The middle phalanx finger-cup allows for rotation while the proximal finger cup is fixed, as per desired kinematic design. Each of the 8-bar mechanisms includes adjustability for different finger lengths. After inspecting the results of the mechanism synthesis, it was apparent that the locations of the end effector **M** as defined for the four hand sizes (Table 5) are very nearly located on a line with respect to link **MY**_1_**Y**_2_. The same is true for the proximal phalange end effector on **PHY**. This fact simplified the mechanical design, allowing for infinite positioning of the finger cups over the full adjustment range. The middle phalanx length adjustment is shown in Figure 6.

**Figure 7:**
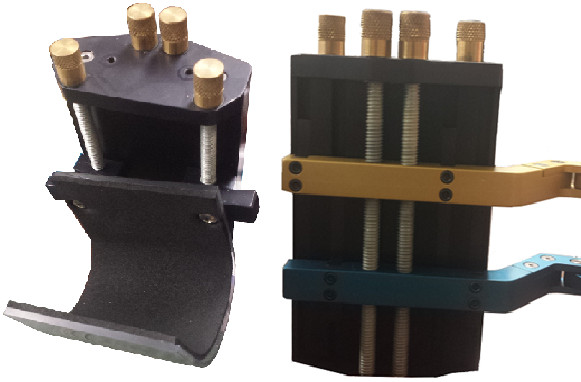
Wrist cuff with height adjustment assembly (left) and mechanism height adjustment (right).

The location of each of the 8-bar mechanisms may be adjusted vertically to align them with the plane of the subjects’ index and middle fingers (see Figure 7). Furthermore, the wrist of the subjects is secured in a wrist cuff, of which the height and angle may be adjusted as necessary for alignment and comfort during gameplay (see Figure 7).

**Figure 8:**
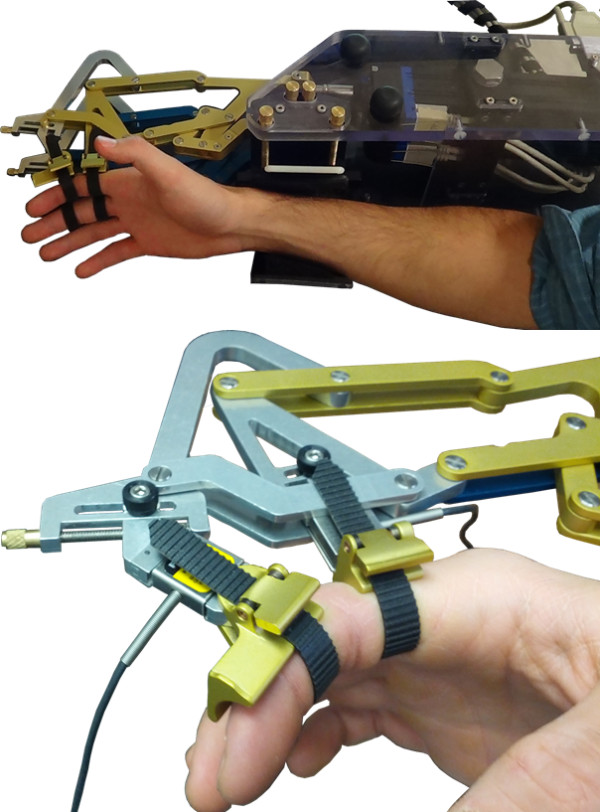
**FINGER assembly.** FINGER robot with two 8-bar finger curling mechanisms and two actuators (top), and close-up of index and middle fingers attached to the robot (bottom). The proximal phalanx finger-cup is fixed at an angle but the middle phalanx is free to rotate.

Each 8-bar mechanism is independently actuated. The two linear actuators are mounted on top of each other with a fixed vertical distance from the base plate while they can freely rotate about an axis normal to it. The detailed specifications of the actuators are explained in the sections that follow. The entire assembly is shown in Figure 8.

### Robotic actuation and performance

#### ***Actuation hardware***

FINGER uses two brushless linear motors (“Servo Tube” actuators, Dunkermotoren STA116-168-S-S03C) to independently actuate the 8-bar finger curling mechanisms. These actuators were chosen for their unique combination of high speed, low friction, and large stroke length. Because they lack any gearing or cables, they exhibit good backdrivability. This is an important feature for robot assisted therapy; the ideal rehabilitation actuator would be able to apply any force at any point during the desired motion, including zero-force, allowing the subject to see the results of their efforts.

This particular model of Servo Tube actuator can produce a continuous force of 26.75 N with a peak of 91.9 N. Current to the actuator is controlled by an amplifier (Copely Controls ACJ-055-09-S), which allows a voltage or PWM setpoint signal. The Servo Tube actuator has built-in Hall Effect sensors and outputs an emulated quadrature encoder position signal of up to 8 microns of resolution. Accelerometers (Analog Devices ADXL325EB) mounted to the end of the actuator rod measures actuator accelerations with a range of ±6 g.

The controller is implemented on a PC using Matlab^®^ xPC Target, with a sampling frequency of 1000 Hz. A National Instruments 6221 DAQ card (16-Bits, 250kS/s) is used to acquire voltage signals from the accelerometers, read the quadrature outputs from the Servo Tube actuators, and send the forces commands to the actuators.

#### ***Control fidelity***

**Figure 9:**
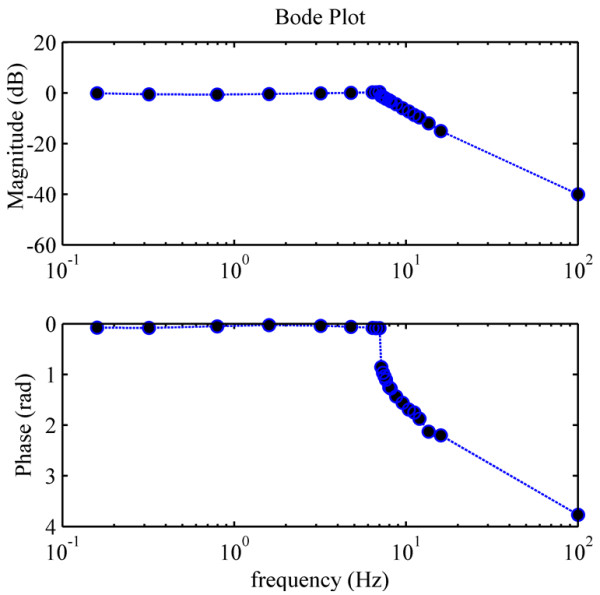
**Bode plots of the robot under PID control.** Reproduced from [22] with permission from IEEE.

To evaluate the control fidelity of FINGER, we conducted a closed loop frequency response test. A Proportional-Integral-Derivative (PID) controller was used to follow desired sinusoidal trajectories with a magnitude of 75% of the range of motion and frequencies from 0.15 to 100 Hz. The PID controller gains, chosen by trial and error, for this test were KP = 8 N/m, KI = 8 N/m∙s and KD = 2 N∙s/m, respectively. The results, shown in Figure 9, show a −3 db magnitude reduction at approximately 8 Hz. The corresponding jump in phase lag indicates the nonlinearity in the system at high speeds.

#### ***Velocity estimation***

Although the built-in position sensor of the Servo Tube actuator has a very high resolution, using a discreet derivative of the position signal can be very noisy, especially at low velocities. In order to obtain a smooth velocity estimate, a Kalman-Filter was designed that uses the actuator’s position signal and an acceleration signal from an accelerometer mounted at the end of the actuator rod. The Kalman-Filter gains were calculated using the Matlab LQR function (Linear Quadratic Regulator). The Kalman-Filter design is similar to the one used in [30].

#### ***Friction compensation***

**Figure 10:**
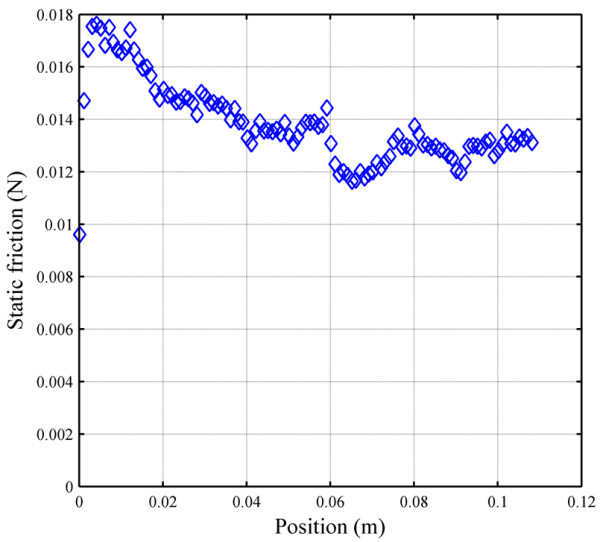
Static friction of the 8-bar finger curling mechanism.

**Figure 11:**
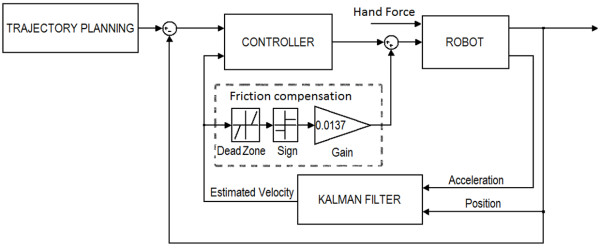
Block diagram of the control system.

Minimizing friction was a top priority during the design and manufacturing of FINGER. This goal guided the mechanism design, manufacturing process, and the selection of bearings and actuators. Figure 10 shows the static friction force for one of the 8-bar mechanisms as a function of actuator stroke. These static friction forces were determined experimentally as the force required by the actuator to move the mechanism from a rest position. Because the position dependency in the static friction is minimal, the average static friction force (0.0137 ± 0.0015 N SD) was used to construct a feedforward friction compensator. Assuming a simple Coulomb friction model, the compensator adds this average friction force along the direction of the estimated velocity. To prevent chattering, the compensator only applied the static force after a minimum velocity magnitude is achieved (see the dotted box in Figure 11).

#### ***Control architecture***

Figure 11 shows the block diagram of the robot control system. The trajectory planning block includes the computer game with predefined desired trajectories, sent to the robot controller. Based on therapeutic preferences, different games can be used or designed as an interface between the subjects and the robot. For each game, the subjects are instructed to move the robot that is attached to their fingers according to the tasks dictated by the game. The robot moves by the combination of subject and actuator forces (Figure 11). The actuator force is a function of the controller type; hence, the controller structure determines how the subject will be assisted by the robot. Various controller types with different characteristics have been used in assistive devices to fulfil different therapeutic hypotheses [13]. The controller used for the testing described herein is a linear Proportional-Derivative (PD) controller, whose gains vary during the gameplay according to an algorithm that will be described in the following sections.

### Pilot testing with individuals with stroke

#### ***Human subjects***

Eleven male and five female volunteers with stroke related motor impairment on the right side participated in the study. The average age of the subjects was 57.8 +/− 12.5 SD and they were 3.3+/−1.8 SD years post stroke. Eight subjects reported that their stroke was ischemic; three reported that their stroke was haemorrhagic; and five did not know. Level of impairment was assessed using both the upper extremity Fugl-Meyer (FM) test and the box and blocks (BB) test [31,32]. For the FM test, a trained therapist asked subjects to perform 33 test movements and scored them 0 (can’t do), 1 (can do partially), or 2 (can do), then summed the scores. For the BB test, subjects moved as many blocks as possible over a divider in a one minute period. The average FM scores for the group were found to be 41.6 ± 15.8 SD out of 66, and average BB scores were found to be 25.1 ± 21.9 (compared to a score of 75.2 ± 11.9 reported in literature for healthy subjects) [32]. Based on these scores, nine of the subjects were classified as highly impaired (FM < 40 & BB < 20), and the remaining seven subjects were classified as moderately impaired. For comparison, four healthy subjects (3 male/1 female, average age 33.5 ± 9.4 SD) were also included in the study. All subjects provided informed consent, and all procedures were approved by the institutional review board at U.C. Irvine.

### Therapeutic game play

**Figure 12:**
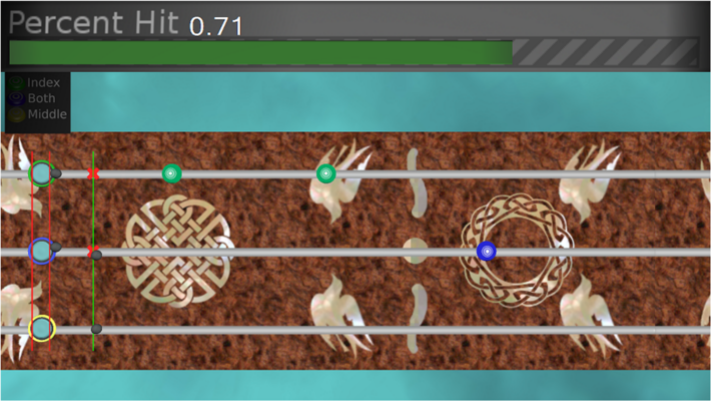
**Screen-shot of the game, which is similar to Guitar Hero^®^. ** The green target was controlled by the index finger, the yellow target by the middle finger, and the blue target by both fingers together. The other two targets were not used. As subjects move a finger, a corresponding dark ball moves along the fret board. The desired locations of the fingers are displayed by fixed circles on the fret board that stream towards the bottom of the screen. To hit a note, the subjects were required to move the ball to the fixed desired position at the time the streaming note passes through it. After hitting a note, the subjects must return the finger(s) to neutral position in order to hit the upcoming notes.

To demonstrate its potential as both a rehabilitative tool and a platform for exploring the factors that promote functional recovery, FINGER was used to test the hypothesis that subjects will be most engaged in the rehabilitation therapy presented to them when they are at their optimal challenge level. To test this hypothesis, FINGER was used to assist subjects in playing a custom-designed game similar to Guitar Hero^®^, which is the third largest video game franchise in history (Additional file 1). Prior to gameplay, the subjects were asked to put their hand in FINGER, and the proximal and middle phalange attachment points were adjusted to finger size until the subjects were able to comfortably curl their fingers through the full range of motion. Additional support under elbow was provided as needed to put the subject’s arm and wrist in a comfortable orientation. This game requires subjects to play along with a song by attempting to hit notes streaming down a visual display as shown in Figure 12. In order to hit these notes, the subjects were required to flex their fingers to a desired angle and stop at the correct time while receiving performance-based assistance from the robot. During the game, subjects were presented with three types of notes corresponding to flexion of the index finger, the middle finger and both fingers together. After successfully hitting a note, the subjects were required to extend their fingers back to a neutral position before the game would credit them with hitting future notes. During extension to the neutral position, subjects received the same amount of assistance as they received during flexion. While subjects attempted to flex their fingers to the correct positions, small dark balls hovering above the fretboard were displayed to provide the subjects with visual feedback of their finger position (see Figure 12). The song used in this experiment was “Happy Together” by the Turtles, and it required 104 notes to be hit over the course of a 160 second game. Timing of the notes was the same during all the experiments and only the level of assistance changed to modulate subjects’ success rates as will be discussed later. Portions of this experiment have been published in conference paper format [33].

#### ***Success rate algorithm***

During the game, FINGER was used to both assist the subjects in completing the desired task and to monitor their performance. Although FINGER can be operated under a variety of control paradigms, this experiment used a PD controller whose gains were intermittently updated by an algorithm which attempts to control the subjects’ probability of hitting notes successfully [34]. Our contention is that by controlling subjects’ success rate, we will be able to control their challenge level. According to the challenge point framework (CPF), determining the optimal challenge level is crucial to optimality of motor learning, particularly in rehabilitation [35]. CPF states there is an ideal amount of information which when presented to the learner will optimize the learning process. In other words, to achieve the best learning rate, the task shouldn’t be too easy or too difficult. This ideal amount of information varies with the skill level of the learner. By controlling the controller gains, we can control the game difficulty and hence the level of challenge the subjects experience, regardless of their impairment level” with “ By changing the feedback gains, we can control the game difficulty and hence the level of challenge the subjects experience, regardless of their impairment level.

Determining the optimal challenge point for a particular task is difficult because it requires measuring long-term learning at a variety of challenge levels in a large number of subjects. However, one determinant of the optimal challenge point is likely effort – i.e. the more engaged a subject is, the more learning will likely occur. Effort can be measured in real-time and thus has the potential to serve as a means to identify when conditions are at least partially conducive for learning. Thus, we studied how effort, quantified by how much force the subjects exerted during the game (see below), varied with success rate.

The success rate algorithm mentioned above works as follows: For each successful note, the algorithm reduced the gains on the corresponding finger by an amount ρ, and for every missed note the gains on the corresponding finger were increased by an amount α∙ρ. As shown in [34], this simple algorithm eventually forces the subjects’ probability of success to converge on a value dependent only on α as shown in Equation 8 below.

(8) P¯=i→∞αα+1

#### ***Experimental protocol***

Subjects were seated in front of a visual display, and the proximal and middle phalanges of their index and middles fingers were securely attached to the end effectors of the FINGER robot. Subjects were then instructed how to play the game and were asked to familiarize themselves with the task by playing through a song at a success rate of 75%. Data from this initial trial were excluded from the final analysis.

After the familiarization task, the robot was used to measure the subjects’ range of motion and maximum isometric force in both flexion and extension. Measurements were taken from the index and middle fingers both individually and together. These measurements were repeated at the end of the experiment. Then subjects were asked to play through the same song twice at each of the three randomly presented success rates (50%, 75%, and 99%).

On a randomly selected subset consisting of roughly 15% of the notes in every song, the robot’s gains were set to a fixed value and the robot was used to block the subject’s movements instead of assisting them. During these blocked trials, the amount of force exerted against the robot was taken as a measure of the subject’s effort in the task. Subject performance during these trials was not used to adapt the robot’s gains, and once the blocked notes passed the control gains were returned to their previous values.

#### ***Data analysis***

The instantaneous success rate at each note was calculated by dividing the number of successful trials within a moving window containing the 25 preceding notes by the size of the window. The peak force applied against the robot during blocked trials was used to quantify subject effort by normalizing it to the subject’s maximum force for the corresponding finger as measured during isometric trials. An unbalanced 2 factor mixed measures ANOVA with repeated measures applied to the success rate variable was used to test the effects of success rate and impairment level on subjects’ effort.

During blocked notes for the index and middle fingers, the robot restricted the motion of both the correct and the incorrect fingers. An estimate of finger individuation was thus obtained by comparing the force generated by the finger that was supposed to move to the force generated by the finger that was not. Forces measured from both fingers were first normalized by their corresponding maximum force values from isometric trials. A measure of individuation was then calculated by dividing the average maximum normalized force applied by the incorrect finger by that of the correct finger. For blocked notes in which the force applied by the incorrect finger was greater than 1.25 times the force applied by the correct finger, it was assumed that the subject accidentally tried to hit the wrong note. Similarly, for trials in which the subjects did not apply any measurable force with either finger, it was assumed the notes were completely missed. These blocked notes were not included in the individuation analysis. An unbalanced three factor mixed measures ANOVA with repeated measures on the finger variable and the success rate variable was used to determine whether finger, success rate, or impairment level had any significant effect on the subject’s individuation value.

## Results

**Figure 13:**
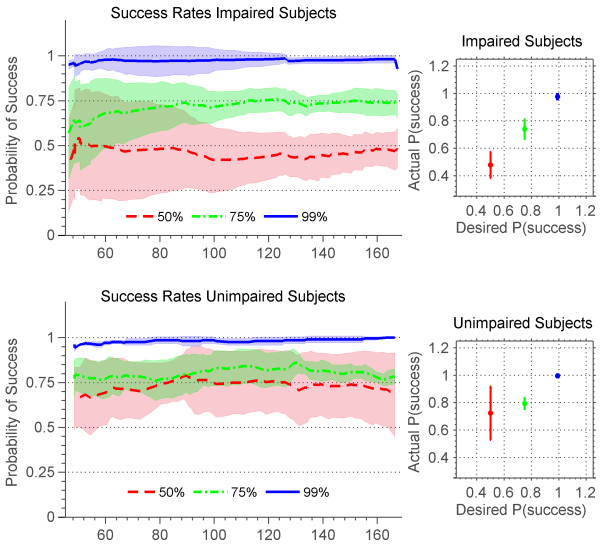
**Actual success rates of stroke and unimpaired subjects.** Actual success rates of stroke (top) and unimpaired (bottom) subjects for songs with desired success rates of 50% (red), 75% (green), and 99% (blue). Plots to the left show time progression of success rates. Lines are the moving window average over subjects and the shaded area is the standard deviation. Plots to the right show mean and standard deviation of desired vs. actual success rates at convergence.

Average probability of success in hitting correct notes during gameplay versus time for the sixteen impaired and the four healthy subjects is shown in Figure 13. At the desired success rates of 50%, 75% and 99% the impaired subjects converged to the average actual success rates of 47.7+/−9.6%, 73.8+/−7.1%, and 97.6+/−1.9%. However, the unimpaired subjects converged to the average actual success rates of 72.2+/−19.5%, 79.3+/−4%, and 99+/−1.1%. This result shows that the algorithm explained in 4.3 is successful in assisting subjects to achieve a desired success rate. It is not surprising that the healthy subjects could achieve success rates higher than algorithm’s desired success rate, because the algorithm doesn’t prevent subjects from hitting more correct notes than desired. In order to effectively challenge the unimpaired subject, the algorithm would need to have been able to make the game more difficult than it would naturally be with the assistance turned completely off. This is not necessary for the impaired subjects, whose reduced neuromuscular ability provided the increased difficulty.

**Figure 14:**
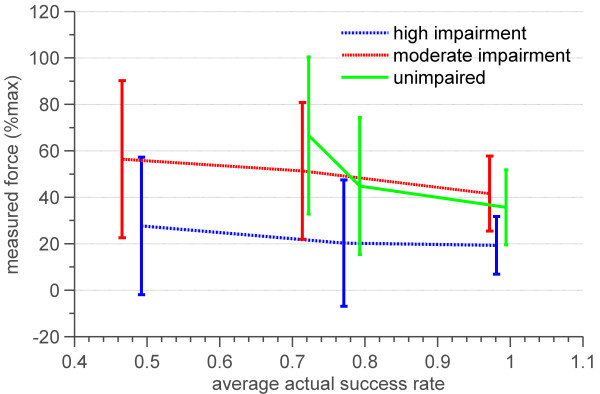
**Average and standard deviation of effort versus average actual success rates for three groups of highly impaired, moderately impaired and unimpaired subjects. ** Effort was quantified as the peak force subjects generated during blocked notes. This peak force was then normalized to each patient’s maximum force generated during isometric test.

We also measured how success rate and impairment level affected the subjects’ effort while playing the game. Success rate was found to have a significant effect on subjects’ effort (p = 0.0024, degrees of freedom = 2). The effects of impairment level on effort, approached but did not achieve significance (p =0.0785, degrees of freedom = 2). As shown in Figure 14, effort decreased when subjects’ success rate increased.

**Figure 15:**
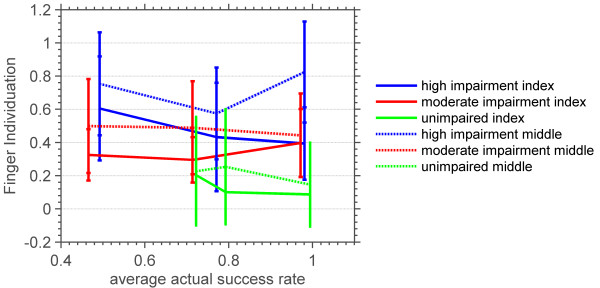
**Average and standard deviation of the index and middle finger individuations versus average actual success rates of three groups of highly impaired, moderately impaired and unimpaired subjects.** Finger individuation was quantified as the ratio of the normalized force generated by the finger that was not supposed to move to that of the finger that was during blocked notes.

**Figure 16:**
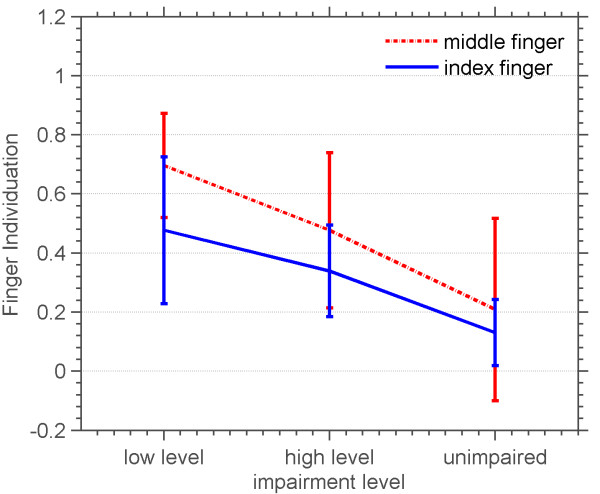
**Average and standard deviation of finger individuation versus impairment level of three groups of highly impaired, moderately impaired and unimpaired subjects.** Average finger individuation across success levels of both middle and index fingers.

Figure 15 shows the effects of impairment level and the finger being used on finger individuation. Both the finger being used and impairment level were found to have a significant effect on finger individuation (p = .0001, degrees of freedom = 1 and p = .0062, degrees of freedom = 2, respectively). As can be seen in Figure 15, individuation scores of the index finger were consistently better than those of the middle finger. This means that when the subject tried to move the index finger, he was more successful at moving the index finger only, as compared to when he tried to move the middle finger. Success rate was not found to have a significant effect on finger individuation, and so we combined data across success levels, resulting in Figure 16, which shows the effect of subjects’ impairment level on finger individuation. Subjects’ with higher impairment had lower individuation ability. The ability to individuate the index finger was higher than the ability to individuate the middle finger.

## Conclusions

This paper described the design and preliminary evaluation of FINGER (Finger Individuating Grasp Exercise Robot). FINGER makes use of individual single degree-of-freedom 8-bar mechanisms to assist patients in making a naturalistic grasping motion with different fingers, together or separately. The kinematic and mechanical design work was guided by the overall goal of creating a robot with high-control fidelity as an instrument for testing and implementing the widest possible range of control strategies. Thus, we paired the lightweight, low-friction mechanism with high-speed and un-geared linear actuators. The resulting robotic mechanism has a closed loop frequency response of approximately of −3 dB at approximately 8 Hz. The fast speed and frequency response of FINGER make it a good candidate for evaluating control algorithms and therapy tasks that require fast movements and/or precise timing.

Another unique feature of FINGER is that, in contrast to most exoskeleton designs that attempt to align the joints of the robot with the joints of the body, the joints of the 8-bar mechanisms of FINGER are kept to the back of the hand and wrist throughout the curling motion. This facilitates easy attachment to the user and stacking of the mechanisms for multiple fingers, and allows for the possibility of applying sensory stimuli to the volar surface of the palm, for example by having individuals grasp real objects with assistance from FINGER.

The physical parameters of the 8-bar mechanism were determined through a mechanism synthesis process that achieved desired end-effector locations using cost function minimization. Four different sets of desired end-effector locations were created to generate a mechanism that could be easily adjusted to accommodate four different hand sizes. Using low-friction bearings and a balanced joint design, we were able to achieve smooth, low-friction 8-bar mechanisms that are easily backdriven. Further design features include finger-to-finder width adjustment, finger length adjustment, and wrist alignment.

Future upgrades to FINGER are currently under development. Possible upgrades and improvements include adding direct force sensing and impedance control, implementing unstructured “assist-as-needed” adaptive control, and adding a thumb exoskeleton mechanism.

The preliminary tests of FINGER showed that it can allow individuals with a range of impairment levels to play an engaging video game similar to Guitar Hero^®^. We used FINGER with a simple gain-adaptation algorithm to test the hypothesis that we can assist subjects as needed in achieving predefined success levels at the game, which we confirmed. We also found that the effort of both high level and low level subjects decreased when their success rate increased; this is consistent with previous observations of slacking when a robotic device over-assists its user [9,10].

According to CPF, there is an intermediate success rate in which learning is maximal. We do not find a success level at which effort was optimal. One possibility is that effort may not decrease unless success is below 50%, the lowest level we tested. Determining the relationship between measures of effort and the optimal challenge point is an important direction for future research.

These tests also demonstrated the ability of FINGER to quantify finger individuation. Using measurements during blocked trials based on patients’ force applied by the wrong finger, we found that patients with higher impairment levels individuated less than those with lower levels of impairment. This result supports the findings in the previous literature on individuation that found that stroke reduced the ability to perform selective individuated finger motions, and specifically that the independence of the middle finger is more impaired than that of the index finger [36,37]. A significant result is that we were able to quantify individuation during the normal course of game play of the game similar to Guitar Hero^®^. The possibility of generating quantitative measures of movement ability while therapy is delivered may increase the frequency at which these measures can be obtained [38].

The results of the preliminary tests with FINGER demonstrate its unique capabilities to study and implement finger therapy after stroke. Additional testing with FINGER may add insight to the effects of success rate on motor learning and finger movement recovery. We also plan to further explore the mechanisms of finger individuation in subjects with impairment due to stroke. Such knowledge will guide the use of FINGER for post-stroke movement therapy.

## Endnote

^a^Guitar Hero^®^ is a trademark of Activision Publishing, Inc.

## Competing interests

The authors declare that they have no competing interests.

## Authors’ contributions

All authors contributed to draft and review the manuscript. ETW, HT, DG, KG and CB contributed to the development and manufacturing of the robot. HT, JBR, DJR and ETW implemented the control design, mechatronics and the game interface. JBR, HT, VC, DG, KG, DJR and ETW carried out the testing of the platform. JBR, DJR, ETW and HT contributed to data analysis. DJR and ETW contributed in the supervision of the research study. All authors read and approved the final manuscript.

## Supplementary Material

Additional file 1 **A video of FINGER being tested. **A lady suffering from stroke is playing the custom designed game similar to GuitarHero^®^ using FINGER. The video game and robot assembly can be seen in the video from two different angles. The MPG video clip can be played using standard video players such as Windows Media Player. Informed consent to publish the video was obtained from the subject. Click here for file

## References

[B1] Roger VL Go AS Lloyd-Jones DM Adams RJ Berry JD Brown TM Carnethon MR Dai S de Simone G Ford ES Heart disease and stroke statistics—2011 update1 Circulation 2011 123 e18 e209 10.1161/CIR.0b013e3182009701 21160056 PMC4418670

[B2] Broeks J Lankhorst G Rumping K Prevo A The long-term outcome of arm function after stroke: results of a follow-up study Disabil Rehabil 1999 21 357 364 10.1080/096382899297459 10503976

[B3] Kwakkel G Kollen BJ Krebs HI Effects of robot-assisted therapy on upper limb recovery after stroke: a systematic review Neurorehabil Neural Repair 2008 22 111 17876068 10.1177/1545968307305457 PMC2730506

[B4] Prange GB Jannink MJA Groothuis CGM Hermens HJ IJzerman MJ Systematic review of the effect of robot-aided therapy on recovery of the hemiparetic arm after stroke J Rehabil Res Develop 2006 43 171 184 10.1682/JRRD.2005.04.0076 16847784

[B5] Lum PS Burgar CG Shor PC Majmundar M Van der Loos M Robot-assisted movement training compared with conventional therapy techniques for the rehabilitation of upper-limb motor function after stroke Arch Phys Med Rehabil 2002 83 952 959 10.1053/apmr.2001.33101 12098155

[B6] van der Lee JH Snels IAK Beckerman H Lankhorst GJ Wagenaar RC Bouter LM Exercise therapy for arm function in stroke patients: a systematic review of randomized controlled trials Clin Rehabil 2001 15 20 10.1191/026921501677557755 11237158

[B7] Platz T Evidence-based arm rehabilitation - a systematic review of the literature Nervenarzt 2003 74 841−+ 14551687 10.1007/s00115-003-1549-7

[B8] Rossini PM Dal Forno G Integrated technology for evaluation of brain function and neural plasticity Phys Med Rehabil Clin N Am 2004 15 263 306 10.1016/S1047-9651(03)00124-4 15029909

[B9] Wolbrecht ET Chan V Reinkensmeyer DJ Bobrow JE Optimizing compliant, model-based robotic assistance to promote neurorehabilitation Neural Syst Rehab Eng, IEEE Trans 2008 16 286 297 10.1109/TNSRE.2008.918389 18586608

[B10] Israel JF Campbell DD Kahn JH Hornby TG Metabolic costs and muscle activity patterns during robotic-and therapist-assisted treadmill walking in individuals with incomplete spinal cord injury Phys Ther 2006 86 1466 10.2522/ptj.20050266 17079746

[B11] Lotze M Braun C Birbaumer N Anders S Cohen LG Motor learning elicited by voluntary drive Brain 2003 126 866 872 10.1093/brain/awg079 12615644

[B12] Kaelin-Lang A Sawaki L Cohen LG Role of voluntary drive in encoding an elementary motor memory 2005 93 1099 1103 10.1152/jn.00143.2004 15456807

[B13] Marchal-Crespo L Reinkensmeyer DJ Review of control strategies for robotic movement training after neurologic injury J Neuroeng Rehab 2009 6 20 10.1186/1743-0003-6-20 PMC2710333 19531254

[B14] Cai LL Fong AJ Otoshi CK Liang Y Burdick JW Roy RR Edgerton VR Implications of assist-as-needed robotic step training after a complete spinal cord injury on intrinsic strategies of motor learning J Neuroscience 2006 26 10564 10568 10.1523/JNEUROSCI.2266-06.2006 PMC6674681 17035542

[B15] Colombo R Pisano F Micera S Mazzone A Delconte C Carrozza MC Dario P Minuco G Robotic techniques for upper limb evaluation and rehabilitation of stroke patients Neural Syst Rehabil Eng, IEEE Trans [see also IEEE Trans Rehabil Eng] 2005 13 311 324 10.1109/TNSRE.2005.848352 16200755

[B16] Krebs HI Palazzolo JJ Dipietro L Ferraro M Krol J Rannekleiv K Volpe BT Hogan N Rehabilitation robotics: performance-based progressive robot-assisted therapy Auton Robot 2003 15 7 20 10.1023/A:1024494031121

[B17] Riener R Lunenburger L Jezernik S Anderschitz M Colombo G Dietz V Patient-cooperative strategies for robot-aided treadmill training: first experimental results Neural Syst Rehabil Eng, IEEE Trans [see also IEEE Trans Rehabil Eng] 2005 13 380 394 10.1109/TNSRE.2005.848628 16200761

[B18] Kahn LE Rymer WZ Reinkensmeyer DJ Adaptive assistance for guided force training in chronic stroke Engineering in Medicine and Biology Society, 2004 EMBC 2004 Conference Proceedings 26th Annual International Conference 2004 1 2722 2725 10.1109/IEMBS.2004.1403780 17270839

[B19] Nowak D The impact of stroke on the performance of grasping: usefulness of kinetic and kinematic motion analysis Neurosci Biobehav Rev 2008 32 1439 1450 10.1016/j.neubiorev.2008.05.021 18582943

[B20] Balasubramanian S Klein J Burdet E Robot-assisted rehabilitation of hand function Curr Opin Neurol 2010 23 661 670 10.1097/WCO.0b013e32833e99a4 20852421

[B21] Cutkosky MR Kao I Computing and controlling compliance of a robotic hand Robotic Autom, IEEE Trans 1989 5 151 165 10.1109/70.88036

[B22] Taheri H Rowe JB Gardner D Chan V Reinkensmeyer DJ Wolbrecht ET Robot-assisted guitar hero for finger rehabilitation after stroke Engineering in medicine and biology society (EMBC), 2012 annual international conference of the IEEE; aug. 28 2012-sept. 1 2012 2012 3911 3917 10.1109/EMBC.2012.6346822 PMC3951853 23366783

[B23] Wolbrecht ET Reinkensmeyer DJ Perez-Gracia A Single degree-of-freedom exoskeleton mechanism design for finger rehabilitation IEEE Int Conf Rehabil Robot: [proceedings] 2011 2011 5975427 10.1109/ICORR.2011.5975427 PMC3951861 22275628

[B24] Heisele B Kressel U Ritter W Tracking non-rigid, moving objects based on color cluster flow Computer vision and pattern recognition, 1997 proceedings, 1997 IEEE computer society conference on; 17–19 jun 1997 1997 257 260

[B25] Book NAS Volume II: a handbook of anthropometric data 1978 NASA Reference: Publication 1024

[B26] Serban I Baritz M Rosca IC Cotoros LD Vlad , Simona , Ciupa , RaduV Statistical Analysis of Anthropometric and Physiologic Performance of the Hand International Conference on Advancements of Medicine and Health Care through Technology IFMBE Proceedings Volume 36 2011 Berlin Heidelberg: Springer 380 383 Chapter: 80

[B27] Dreyfuss H Tilley A Measure of man and woman: human factors in design 1993 New York City, NY: Watson-Guptill Publications

[B28] Sandor GN Erdman AG Advanced mechanism design: analysis and synthesis: vol. 2 1984 Englewood Cliffs: NJ, Prentice-Hall

[B29] Soh GS McCarthy JM The synthesis of six-bar linkages as constrained planar 3R chains Mech Mach Theory 2008 43 160 170 10.1016/j.mechmachtheory.2007.02.004

[B30] Wolbrecht ET Reinkensmeyer DJ Bobrow JE Pneumatic control of robots for rehabilitation Int J Robot Res 2010 29 23 38 10.1177/0278364909103787

[B31] Fugl-Meyer AR Jaasco L Leyman L Olsson S Steglind S The post-stroke hemiplegic patient Scand Journal Rehab Med 1975 7 13 31 1135616

[B32] Mathiowetz V Volland G Kashman N Weber K Adult norms for the box and block test of manual dexterity Am J Occup Ther 1985 39 386 391 10.5014/ajot.39.6.386 3160243

[B33] Taheri H Rowe JB Gardner D Chan V Reinkensmeyer DJ Wolbrecht ET Robot-assisted guitar hero for finger rehabilitation after stroke 2012 San Diego, CA: Engineering in Medicine and Biology Society (EMBC), 2012 Annual International Conference of the IEEE 10.1109/EMBC.2012.6346822 PMC3951853 23366783

[B34] Spencer SJ Movement training and post-stroke rehabilitation using a six degree of freedom upper-extremity robotic orthosis and virtual environment 2012 Irvine: University of California

[B35] Guadagnoli MA Lee TD Challenge point: a framework for conceptualizing the effects of various practice conditions in motor learning J Motor Behav 2004 36 212 224 10.3200/JMBR.36.2.212-224 15130871

[B36] Lang CE Schieber MH Differential impairment of individuated finger movements in humans after damage to the motor cortex or the corticospinal tract J Neurophysiol 2003 90 1160 1170 10.1152/jn.00130.2003 12660350

[B37] Lang CE Schieber MH Reduced muscle selectivity during individuated finger movements in humans after damage to the motor cortex or corticospinal tract J Neurophysiol 2004 91 1722 1733 10.1152/jn.00805.2003 14668295

[B38] Zariffa J Kapadia N Kramer J Taylor P Alizadeh-Meghrazi M Zivanovic V Albisser U Willms R Townson A Curt A Relationship between clinical assessments of function and measurements from an upper-limb robotic rehabilitation device in cervical spinal cord injury Neural Syst Rehabil Eng, IEEE Trans 2011 341 350 10.1109/TNSRE.2011.2181537 22203726

